# Bluefin tuna reveal global patterns of mercury pollution and bioavailability in the world's oceans

**DOI:** 10.1073/pnas.2111205118

**Published:** 2021-09-13

**Authors:** Chun-Mao Tseng, Shin-Jing Ang, Yi-Sheng Chen, Jen-Chieh Shiao, Carl H. Lamborg, Xiaoshuai He, John R. Reinfelder

**Affiliations:** ^a^Institute of Oceanography, National Taiwan University, Taipei 10617, Taiwan;; ^b^Ocean Sciences Department, Institute of Marine Sciences, University of California, Santa Cruz, CA 95064;; ^c^Department of Environmental Sciences, Rutgers University, New Brunswick, NJ 08901

**Keywords:** bluefin tuna, mercury bioaccumulation, bioavailability, ocean pollution, mercury accumulation rate

## Abstract

Bluefin tuna (BFT), highly prized among consumers, accumulate high levels of mercury (Hg) as neurotoxic methylmercury (MeHg). However, how Hg bioaccumulation varies among globally distributed BFT populations is not understood. Here, we show mercury accumulation rates (MARs) in BFT are highest in the Mediterranean Sea and decrease as North Pacific Ocean > Indian Ocean > North Atlantic Ocean. Moreover, MARs increase in proportion to the concentrations of MeHg in regional seawater and zooplankton, linking MeHg accumulation in BFT to MeHg bioavailability at the base of each subbasin's food web. Observed global patterns correspond to levels of Hg in each ocean subbasin; the Mediterranean, North Pacific, and Indian Oceans are subject to geogenic enrichment and anthropogenic contamination, while the North Atlantic Ocean is less so. MAR in BFT as a global pollution index reflects natural and human sources and global thermohaline circulation.

## Significance

Bluefin tuna (BFT) is an apex predatory, long-lived, migratory pelagic fish that is widely distributed throughout the world's oceans. These fish have very high concentrations of neurotoxic methylmercury (MeHg) in their tissues, which increase with age. Our study shows that Hg accumulation rates (MARs) in BFT as a global pollution index can reveal global patterns of Hg pollution and bioavailability in the oceans and further reflect both natural and anthropogenic emissions and regional environmental features. Overall, MARs provide a means to compare Hg bioavailability among geographically distinct populations of upper trophic level marine fish across ocean subbasins, to investigate trophic dynamics of Hg in marine food webs, and furthermore, to improve public health risk assessments of Hg exposure from seafood.

As the world’s largest tuna (up to 300 cm and 600 kg), bluefin tuna (BFT) is an apex predatory and long-lived (>30 y) fish (*SI Appendix*, Table S1) (1). Increasing worldwide demand for BFT has led to overfishing and severe depletion of stocks (2). At the same time, rising human consumption of BFT may increase the risk of exposure to highly bioaccumulated contaminants, including Hg (3). Since BFT accumulate Hg through the dietary transfer of MeHg, nearly all the Hg (total Hg, THg) in its muscle tissue is neurotoxic MeHg (4–6). Efficient trophic transfer leads to MeHg concentrations in BFT that are up to 10^8^ times higher than those in seawater (3–7). As a result, tissue Hg concentrations in BFT often exceed threshold human consumption guidelines for large predatory fish of 1 µg ⋅ g^−1^ wet weight (w.w.) set by the US Food and Drug Administration and the World Health Organization (8). Predictive models of Hg levels in BFT are therefore needed to safeguard public health.

**Fig. 1.**
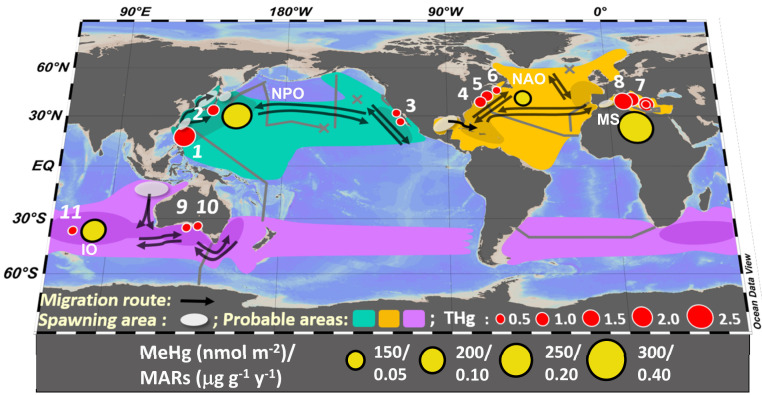
Global distributions, spawning areas, migration routes, and average THg concentrations (μg ⋅ g^−1^ w.w.) of the three BFT species and their subpopulations. Cruise tracks and vertical profile stations where seawater Hg concentrations were measured are indicated by gray lines and crosses. BFT capture locations are indicated by numbers: 1 and 11, this study; 2 and 6, ref. 51; 3 refs. 4, 41; 4, ref. 11; 5, ref. 52; 7, refs. 5, 16, 17; 8, ref. 18; 9, ref. 53; and 10, ref. 54. High density (dark shaded) and probable (light shaded) spatial distributions of BFT in the North Pacific (*Thunnus* *orientalis,* Pacific BFT in viridian), North Atlantic (*Thunnus* *thynnus,* Atlantic BFT in orange), and Southern Indian Oceans (*Thunnus* *maccoyii,* Southern BFT in purple) are shown (IUCN (International Union for Conservation of Nature). 2019, http://www.iucnredlist.org). MARs in BFT (yellow circles) in relation to methylmercury (MeHg) inventories in the world's oceans are also shown (see *MARs in BFT Reveal Global Patterns of Pollution and Bioavailability*).

The three species of BFT include the Southern BFT (*Thunnus maccoyii*), Pacific BFT (*Thunnus orientalis*), and Atlantic BFT (*Thunnus thynnus*), whose migratory routes are confined to southern hemisphere oceans (including the Indian Ocean, IO), the North Pacific Ocean (NPO), and the North Atlantic Ocean (NAO)/Mediterranean Sea (MS), respectively (Fig. 1 and *SI Appendix*, Table S1). Although they have slightly different biological and ecological characteristics (*SI Appendix*, Table S1), being closely related, the three BFT species have similar pelagic migration and feeding behaviors. BFT are oceanic epipelagic fish that commonly spend most of their time (>90%) in waters shallower than 200 m, although they can dive to depths of several hundred meters (*SI Appendix*, Table S1). While their spawning is confined to specific locations and seasons, BFT engage in opportunistic feeding across vast areas of the ocean subbasin each inhabits (*SI Appendix*, Table S1). Since Hg levels in marine fish reflect concentrations in their prey and ultimately local seawater (9), the longevity and migratory and epipelagic behavior of BFT make them excellent integrators of Hg levels in their respective ocean domains, which are known to vary globally (7).

Various species of tuna have been proposed as bioindicators of temporal changes in oceanic Hg pollution (9–13). However, direct comparisons of fish tissue Hg concentrations across space and time are difficult because in addition to taxonomic differences, Hg levels in marine fish are affected by age, size, trophic position, and prey type and abundance, which vary according to local and global environmental conditions. For example, it has been reported that Hg concentrations in various species of tuna are increasing in the central PO (10), decreasing in the NAO (11), or display significant interannual variability in the Southwestern PO (12). However accurate, the utility of such findings as the basis of interocean basin comparisons of Hg pollution may be questioned due to differences among taxonomic groups and temporally uneven or insufficient sample sizes. To eliminate such limitations, and to provide a standard basis of comparison to examine spatial and temporal trends of Hg pollution across the world's oceans, we propose to compare mercury accumulation rates (MARs), defined as a change in muscle Hg concentration per unit change in either size/weight or age, among the three species of BFT from four different ocean subbasins.

In view of the above, the primary goal of this study was to evaluate how Hg bioaccumulation in BFT varies on a global scale and whether such variation corresponds with levels of Hg pollution across ocean basins. To examine recent global patterns of Hg pollution in the world's oceans (including the 22-y period from 1998 to 2019), we first calculate the apparent MAR as the slope of the relationship between muscle THg concentrations and age based on new measurements of Pacific BFT from the western NPO caught in 2017 to 2018 and Southern BFT from the IO caught in 2018 to 2019, as well as previously available results for fish from the NAO and MS collected from 1998 to 2017 (Fig. 1 and *SI Appendix*, Fig. S1). Furthermore, observed differences in MARs in BFT were examined in relation to the variation of MeHg concentrations in water and plankton, obtained from previous studies, in surface (0 to 150 m) and thermocline layers (150 to 1,000 m) and in water column inventories (nmol m^−2^, integrated from the surface to 1,000 m, covering all the foraging depths of BFTs) across four ocean subbasins. Finally, based on a detailed compilation of the global patterns of Hg cycling and fluxes in the ocean, we examine relationships between Hg accumulation in BFT and human sources and global thermohaline circulation.

## Results

### THg Levels in Pacific BFT and Southern BFT Muscles.

Our results include THg concentrations in adult Pacific BFT from the NPO (≥5 y old) and in >4 y old Southern BFT from the IO. The adult Pacific BFT from the NPO we analyzed spanned wide ranges of size and body mass (*SI Appendix*, Table S2), and based on fork length (FL) (14) (*SI Appendix*), varied in age from 5 to 27 y old (∼85% in age groups of 5 to 15 y). THg levels in the muscle tissue of these Pacific bluefin tuna (Pacific BFT) ranged from 0.49 to 5.65 µg ⋅ g^−1^ w.w. (mean = 2.00 ± 0.83 µg ⋅ g^−1^ w.w., *n* = 261), ∼94% of which exceeded the safe consumption guideline of 1 µg ⋅ g^−1^ w.w. The Southern bluefin tuna (Southern BFT) from the IO we analyzed were 2 to 15 y old (ref. 15; reference *SI Appendix*) and had muscle tissue THg concentrations of 0.20 to 1.85 μg ⋅ g^−1^ w.w. (mean = 0.42 ± 0.24, *n* = 83; ∼2% of which exceeded 1 µg ⋅ g^−1^ w.w.).

## Discussion

### Variation in THg Levels among BFT Subpopulations.

**Fig. 2.**
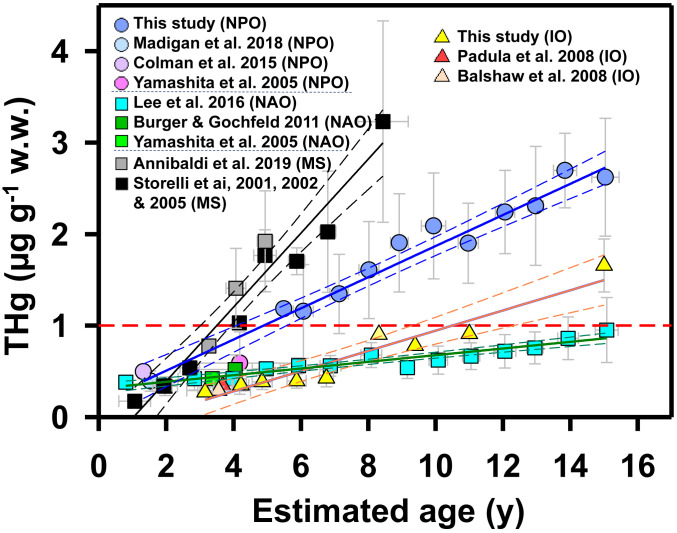
Relationships between THg concentrations and fish age in ≤15-y-old BFT from the NPO, the MS, the NAO, and the IO. The dashed red line denotes World Health Organization food safety limit of 1 µg ⋅ g^−1^ w.w. Values are means ± 1 SD. Regression lines (solid lines) with 95% CIs (dashed lines) are plotted for BFT from each basin (*SI Appendix*, Table S3 for regression statistics).

Based on these and previously published results, and as shown for other tuna species (13), mean THg concentrations in BFT muscle varied between and within ocean basins (Fig. 1 and *SI Appendix*, Table S2). Indeed, among all four populations examined, mean concentrations of THg in BFT declined in the order NPO > MS > NAO > IO. This differs from previously reported trends, including those suggesting that BFT from NAO have higher average Hg concentrations than BFT from all other oceans (1). However, due to differences in the age and size of fish across studies, direct comparisons of mean fish tissue concentrations may not accurately represent the relative tendencies of geographically distinct populations of BFT to accumulate Hg. As in other marine predatory fish, concentrations of Hg in BFT increase with fish age and size (Fig. 2 and *SI Appendix*, Fig. S2) consistent with expected allometric relationships (5, 11, 16–18). Since BFT grow more or less continuously throughout their lives, increasing Hg concentration with age primarily reflects the slow turnover of Hg in fish muscle (19) and the small extents of in vivo demethylation in fish (20). Atlantic BFT from the MS and Southern BFT from the IO that have been analyzed for Hg concentration are all ≤15 y old. In addition, most (∼85%) of the Pacific BFT we analyzed were ≤15 y old, and most of the fish older than 15 y (growth rate ∼1 cm ⋅ y^−1^) were at the upper limit of the FL–age model and therefore of uncertain age (*SI Appendix*, Fig. S3). To account for differences in fish age and size across studies and geographical domains, and to eliminate the influence of very old/large fish, we used age-based MARs estimated for ≤15-y-old fish to scale Hg accumulation among BFT subpopulations from different ocean subbasins.

### MARs in BFT Reveal Global Patterns of Pollution and Bioavailability.

**Fig. 3.**
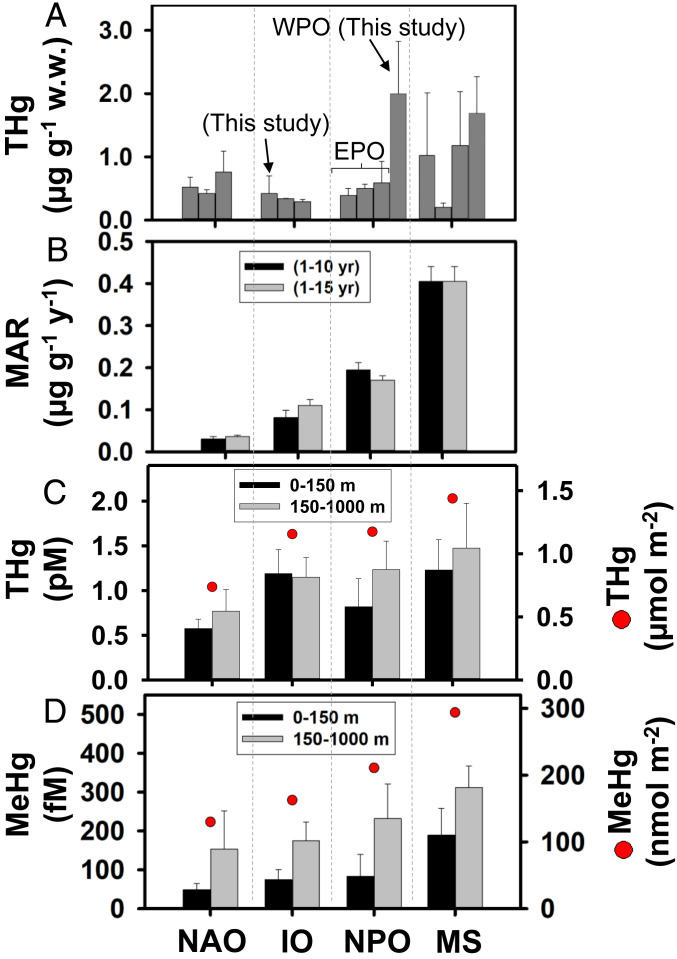
Global comparison of (*A*) THg concentrations and (*B*) MARs in BFT, and average concentrations and water column inventories (0 to 1,000 m) of (*C*) THg and (*D*) methylmercury (MeHg) in the surface ocean (0 to 150 m) and thermocline layer (150 to 1,000 m) of the NAO, the IO, the eastern PO (EPO), western PO (WPO), NPO, and the MS. Sources for BFT concentrations are in Fig. 2 and *SI Appendix*, Table S2. Seawater concentrations and water column inventories are from *SI Appendix*, Table S8. Values are means ± 1 SD.

Our analysis shows that age-based MARs estimated for ≤15-y-old BFT binned into 1-y age classes varied significantly among BFT populations from four ocean subbasins (Fig. 2 and *SI Appendix*, Tables S3 and S4). Overall, the highest age-based MAR for BFT was observed in the MS, followed by the NPO, the IO, and lastly the NAO (Fig. 3). In Atlantic BFT from the MS, MARs were 2 to 4 times higher than those in Pacific BFT from the NPO and Southern BFT from the IO and 8 to 10 times higher than those in Atlantic BFT from the NAO (*SI Appendix*, Table S3). The same global trend in MARs was found when BFT of all ages were included, although the inclusion of older fish resulted in somewhat higher MARs for the NAO and lower MARs for the NPO (*SI Appendix*, Table S3). Please note that MARs for two samples of Atlantic BFT from the MS, one collected in 2000 to 2005 and the other in 2019 (5, 16–18), were not significantly different (one-way ANOVA, *P* > 0.1) and were both the highest among the four ocean basins we examined (Fig. 2 and *SI Appendix*, Fig. S2). Thus, while Hg concentrations in BFT within the same region might vary slightly over two decades, such variation does not affect the global patterns of MARs we observed.

**Fig. 4.**
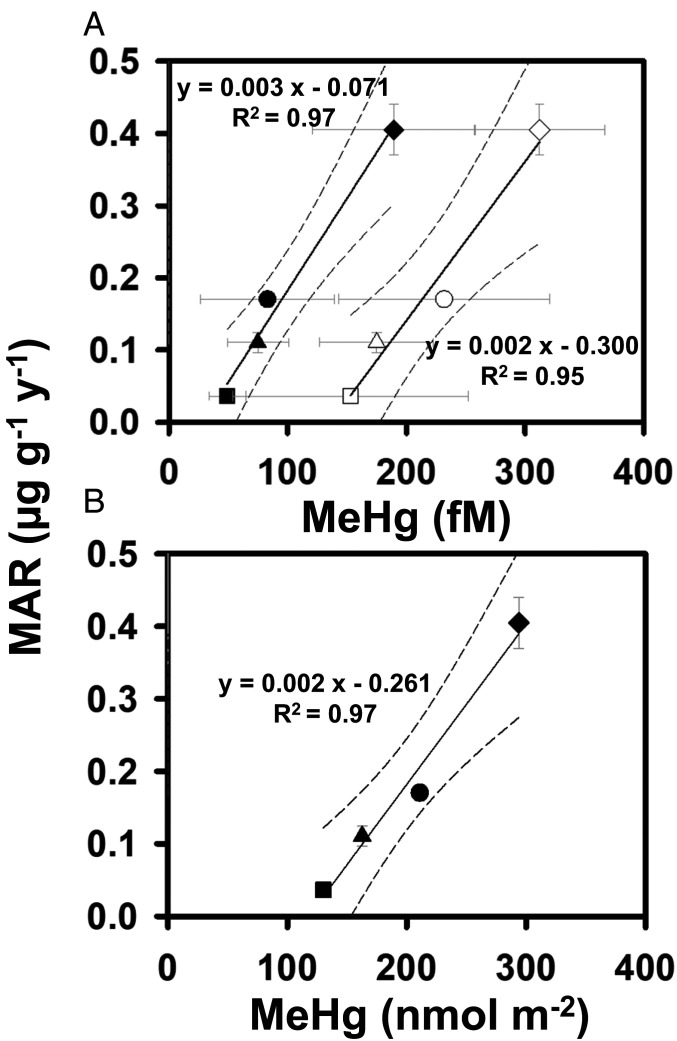
Relationships between MARs in BFT and (*A*) methylmercury (MeHg) concentrations in surface (0 to 150 m, solid black) and thermocline layers (150 to 1,000 m, open symbols) and (*B*) water column inventories (0 to 1,000 m) of MeHg across four ocean subbasins, the NAO (square), the IO (triangle), the NPO (circle), and the MS (diamond). Errors are ±1 SD. Regression lines (solid lines) with 95% CIs (dashed lines) are plotted.

**Fig. 5.**
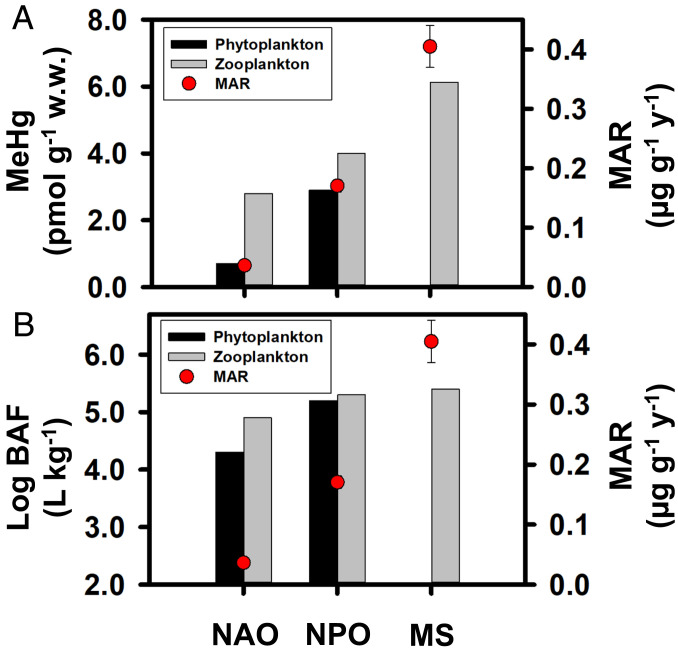
Comparisons of MARs (average ±1 SD) in BFT with mean (*A*) concentrations and (*B*) bioaccumulation factors (BAF) of methylmercury (MeHg) in marine phytoplankton and zooplankton in three ocean basins. BAF is the MeHg concentration (pmol ⋅ kg^−1^, w.w.) in an organism divided by the dissolved concentration of MeHg (pmol ⋅ L^−1^) in seawater.

Across four ocean subbasins, Hg accumulation rates in BFT were significantly and positively correlated with seawater Hg concentrations, especially MeHg and its water column inventory (nmol ⋅ m^−2^) integrated from the surface to 1,000 m (Figs. 3 and 4; *P* < 0.001). The relationship between MeHg inventories from the surface to 1,000 m and MARs in BFT may be particularly strong (*R*^2^ = 0.97) because the inventories integrate possible differences in vertical foraging habits of BFT that may influence their Hg concentrations. Furthermore, MARs in BFT from the MS, NPO, and NAO were also positively correlated with concentrations and bioaccumulation factors of MeHg in phytoplankton and zooplankton from each ocean basin (*P* < 0.001; Fig. 5 and *SI Appendix*, Tables S5 and S6). Such correlations provide evidence linking dissolved MeHg to its accumulation in apex predators at the top of oceanic food webs, which is expected but rarely observed directly due to inadequate sample sizes. This further implies that Hg concentrations in BFT prey also vary with MeHg concentrations in seawater (9, 21).

In addition to the concentration of MeHg, other eco-physiological factors such as temperature, diet, food web structure, migration pattern, and growth rate could also alter Hg accumulation rates in BFT (*SI Appendix*, Table S6). Those factors are mostly controlled by water temperature in different geographic locations. Dell'Apa et al. (22) examined many temperature-influenced processes in BFT. They found that the effect of water temperature on BFT is mainly on larval survival and less on adult growth. In addition, growth rates of BFT are less sensitive to temperature changes due to their ability to 1) maintain stable body temperature, and 2) adjust the timing and range of their foraging and migration as water temperature changes (23, 24). The spatial distributions of BFT populations are, however, driven by the availability of food, not by the temperature of seawater. Growth rates, which integrate many eco-physiological properties such as temperature and food web structure/efficiency, were not correlated (*P* = 0.7) with Hg accumulation rates in the four BFT populations examined. However, Hg accumulation rates in BFT populations are strongly positively correlated with the concentration of MeHg in the water column and in planktonic organisms at the base of each basin's food web (Figs. 4 and 5 and *SI Appendix*, Table S6). This is consistent with recent model results, which indicate that most (60 to 75%) of the variation in MeHg concentrations in Atlantic BFT is due to variation in the concentration of MeHg in seawater (21). Our observations therefore indicate that the interbasin variation in Hg accumulation rates among BFT populations is primarily controlled by the bioavailability of Hg in the waters in which they feed, and other eco-physiological traits likely play secondary roles.

### Global Variation of Hg Accumulation Rates in BFT.

Across ocean basins, Hg accumulation rates in BFT and upper water column MeHg inventories correspond closely to Hg inputs to each ocean basin (Fig. 1 and *SI Appendix*, Fig. S5). The ocean basin with the highest MAR for BFT is the MS. This indicates that the effects of regional contamination and/or the production and food web bioavailability of MeHg are greater in the MS compared to other ocean subbasins. Hg contamination of the MS ecosystem has been a concern for decades (25) and is the result of both anthropogenic and natural volcanic activities. According to the current Hg budgets (26, 27), Hg evasion outputs from the MS are nearly equal to inputs from atmospheric, riverine, and geogenic sources (*SI Appendix*, Figs. S4 and S5 and Table S7). Relatively high fluxes of Hg to this nearly landlocked marginal basin, which experiences little exchange with the open North Atlantic results in lower variability of THg concentrations in the surface waters and thermocline of the MS than in other ocean basins (*SI Appendix*, Table S8). In addition, as a result of high seawater temperatures and metabolic (oxygen consumption) rates in strongly stratified and primarily oligotrophic surface waters (28, 29), the MS may support high Hg methylation rates coupled to the remineralization of small, slowly sinking nano- and picophytoplankton cells (30). This would result in higher seawater concentrations of MeHg (*SI Appendix*, Table S8) and higher levels of Hg in Mediterranean BFT (5, 16–18) than in other ocean basins. Please note that although a few Atlantic BFT of Mediterranean origin have been identified in the western Atlantic's Slope Sea (31) and in the Denmark Strait east of Greenland (32), tagging studies show that Atlantic BFT from the MS generally do not migrate throughout the NAO (33, 34) and appear to spend nearly all of their time in the Mediterranean (35). Atlantic BFT caught in the MS therefore primarily reflect MS Hg levels.

The ocean subbasin with the second-highest MAR for BFT, as revealed by our results, is the NPO, which also has the second-highest seawater MeHg concentrations and inventories among the world's oceans (Fig. 3 and *SI Appendix*, Table S8). The NPO is downwind of East Asia, which is the world's largest emitter of Hg to the atmosphere (Fig. 1 and *SI Appendix*, Fig. S5) (36). Additionally, the western NPO receives a substantial load of Hg from the discharge of polluted East Asian rivers such as Changjiang and Zhujiang (37, 38). Ignoring vertical fluxes within the ocean, atmospheric deposition and riverine inputs exceed volatilization and result in a total net input flux of over 55 nmol ⋅ m^−2^ ⋅ y^−1^ to NPO surface waters (*SI Appendix*, Fig. S5). Since the majority of the mercury in contemporary atmospheric and riverine inputs is anthropogenic (39), the high MAR in BFT from the NPO appears to be largely a consequence of human-caused Hg loadings. This is consistent with previous reports showing increasing concentrations of Hg in Pacific yellowfin tuna over the past decade (10) and modeling results showing increasing Hg in the NPO as a whole (37). Given their breeding and migration patterns (40) (Fig. 1), Pacific BFT may accumulate anthropogenic Hg in waters off the eastern coast of Taiwan, the coasts of Japan, as well as among the Ryukyu Islands and the Philippine Sea, which have experienced increasing Hg concentrations in recent decades (37, 38).

Tissue Hg concentrations are available for Pacific BFT from the eastern NPO, but these include only juvenile fish with ages under 5 y (4, 41). Juvenile Pacific BFT that migrate to the eastern NPO eventually return back to western NPO waters near Japan, Taiwan, and the Philippines for spawning by age 5 to 7 (40, 42) (Fig. 1). Resident or migrant adult Pacific BFT generally travel northeast to feeding grounds east of Japan after spawning and spend the rest of their lives across a broad area of the NPO (43). Therefore, the bioaccumulation of Hg in the Pacific BFT we analyzed for this study likely reflects the Hg content of seawater across wide areas of the North Pacific.

The MARs for the North Atlantic population of Atlantic BFT that we estimated from the results of Lee et al. (11) were considerably lower than those for BFT from the MS and NPO and were two to three times lower than those for Southern BFT from the IO. This is consistent with observations of lower seawater concentrations and inventories of Hg in the NAO than in the NPO, MS, and IO (Figs. 2–4 and *SI Appendix*, Table S8). As a result of lower atmospheric deposition and riverine inputs from North America and Europe, net inputs of Hg to the NAO (∼10 nmol ⋅ m^−2^ ⋅ y^−1^) are also lower than those in the other subbasins (*SI Appendix*, Fig. S5). Lee et al. (11) suggested that declines in Hg concentrations of Atlantic BFT between 2004 and 2012, which corresponds to a period of declining atmospheric Hg concentrations over the NAO (44) and in seawater of the eastern NAO and western MS (45), were tied to reductions in anthropogenic Hg emissions in North America and Europe (44). Lower inputs of inorganic Hg may account for the lower upper-ocean (1,000 m) water column inventory of MeHg in the NAO than in the IO and NPO (Fig. 3*D* and *SI Appendix*, Fig. S5), but other factors related to ocean circulation and biogeochemical processes may also contribute to these differences. For instance, little upwelled or anthropogenic MeHg from the NPO and the IO is expected to return to the NAO via global thermohaline circulation (*SI Appendix*, Fig. S5).

Our observation that MARs for Southern BFT from the IO were higher than those for Atlantic BFT from the NAO is somewhat surprising given that there are fewer terrestrial sources and anthropogenic emissions of Hg to oceans in the southern hemisphere, where open ocean accounts for ∼81% of the surface area, than to the NPO and NAO (46). However, while the net input of Hg to the IO (∼18 nmol ⋅ m^−2^ ⋅ y^−1^) is much lower than that to the NPO (55 nmol ⋅ m^−2^ ⋅ y^−1^), it is nearly double that to the NAO (10 nmol ⋅ m^−2^ ⋅ y^−1^) (*SI Appendix*, Fig. S5). While MeHg concentrations are relatively low in the northern IO, including the oxygen minimum zones of the Arabian Sea (47), surface waters of the southern IO may accumulate upwelled MeHg or recent anthropogenic Hg primarily from the western equatorial PO via the Indonesian Throughflow (*SI Appendix*, Fig. S5).

## Conclusions

We found significant variation in MARs among four geographically distinct populations of BFT (Fig. 1 and *SI Appendix*, Fig. S5). This variation may well reflect different levels of Hg pollution or natural sources and different mechanisms of MeHg production and transport associated with contrasting ecological structures and circulation patterns across ocean subbasins. MARs in BFT and potentially other large marine predators can therefore be used as global indices of Hg concentrations and bioavailability in the world's oceans. Additionally, the MAR index presents a possible refinement of safe consumption guidelines, which currently treat all fish of a certain species the same. For example, the MAR along with information on the fish size (e.g., FL, weight) and the ocean basin of capture could be used to estimate whether or not Hg concentrations in an individual fish exceed food safety limits and safe consumption rates (*SI Appendix*). Thus, variations in Hg accumulation among populations of marine fish from distant ocean basins can be used to study global Hg contamination, manage marine fisheries, and safeguard human health.

## Materials and Methods

### Sample Information.

Muscle tissue was sampled from Pacific BFT (*n* = 261) captured by Taiwan longline fishing vessels in the western NPO (18° to 21° N, 120° to 127° W) from April to July in 2017 and 2018 during the spawning period and from Southern BFT (*n* = 83) in the southern IO (32° to 41° S, 67° to 80° W) from March to July in 2018 and 2019 (*SI Appendix*, Fig. S1). Samples were taken from whole fish landed at the fishing ports in Taiwan, and the FLs of the individual Pacific BFT and Southern BFT (the measurement from the tip of the rostrum to the fork of the tail; cm) were measured by the observer. Muscle tissue was taken from the hypaxial dorsal musculature and immediately frozen at −5 °C and then stored at −20 °C once returned to the laboratory. Muscle tissue was subsequently freeze-dried for 96 h and then homogenized before Hg analysis.

The sample set included 261 Pacific BFT and 83 Southern BFT with males and females occupying the same size range. FL and body mass of the 261 Pacific BFT ranged from 182 to 266 cm (average ± SD: 217 ± 19) and 113 to 392 kg (212 ± 61) and of the 83 SBFT from 85 to 166 cm (120 ± 16) and 13 to 96 kg (28 ± 13), respectively. The age of the captured Pacific BFT was estimated from FL by an empirical age-length equation that was directly developed from otoliths of the Pacific BFT population in the southern spawning ground near Taiwan (14). Estimation of age in the Pacific BFT FL greater than 250 cm, circa >25 y, is highly uncertain; as a result, the Pacific BFT estimated >25 y old are all grouped as 27 for the purpose of analysis. The age of the Southern BFT was then from 2 to 15 y old estimated by the age-length key for the Southern BFT population in southern IO and obtained by direct estimation from otoliths as well (15).

### Mercury Analysis.

In this study, THg analysis was carried out after quantitative recoveries of all Hg from lyophilized muscle tissues (0.02 to 0.05 g) by microwave-assisted acid bomb digestion with a 4:1 (volume:volume) mixture of HNO_3_ (4 mL) and HCl (or H_2_SO_4_, 1 mL) (48, 49). Tuna THg concentrations were then measured using a flow injection THg analyzer (THg-FIMA), which is a dual purge-and-trap system combining aqueous reduction with stannous chloride (SnCl_2_), two-stage gold (Au) amalgamation, and thermal desorption together with cold vapor atomic fluorescence spectrometry (50). THg concentrations represented MeHg concentrations in BFT in this study since MeHg accounts for nearly all mercury in BFT in Pacific BFT from the western PO (WPO) (*SI Appendix*). THg concentrations in BFT were expressed in μg ⋅ g^−1^ w.w. through the correction of water content (Pacific BFT: 73 ± 4%, ∼61 to 81%; Southern BFT: 71 ± 4%, ∼63 to 80%) from the dry weight (d.w.) concentration. Detailed information regarding the analytical performance and data quality with recoveries of Hg from the two certified biological reference materials (TORT-2, lobster hepatopancreas, 0.270 ± 0.001 μg ⋅ Hg ⋅ g^−1^ d.w.; IAEA (International Atomic Energy Agency)-436, tuna fish, 4.19 ± 0.36 μg ⋅ g^−1^ d.w.) was described in *SI Appendix*.

### Global Hg Literature Review in BFT and Oceans.

A focus of this work is a comparison of relationships between Hg and fish age or size in a variety of locations worldwide. Our preferred metric for this comparison was the MAR for each fish population. We thus collected published total Hg concentrations in muscle tissue of BFT and accompanying metadata, including capture date and location, FL, fish weight, and age. Mean concentrations of Hg in size/weight groups of BFT were included if raw individual data were not reported. In some cases, data were reported graphically, and Web Plot Digitizer (https://automeris.io/WebPlotDigitizer) was used to extract the values of Hg, fish size, age, etc. All data were expressed in μg ⋅ g^−1^ w.w. basis for analyses.

The four ocean regions where BFT were captured included the following: NPO (WPO and EPO) (4, 41, 51), NAO (11, 51, 52), MS (5, 16–18), and IO (53, 54). The most complete datasets, in which BFT THg concentrations were reported for a wide range of fish size and mass, were from the NAO (age 1 to 29) and WPO (1 to 27, this study). Those BFTs whose THg levels were available in small-sized fish (age 1 to 15) were from the MS and the southern IO (this study with two previous studies). Summaries of previously published BFT THg concentrations with associated metadata and Hg concentrations in seawater and plankton for various ocean regions are provided in *SI Appendix*, Tables S2, S5, S6, and S8. Conversions from BFT body weight to length or vice versa or to age based on the growth equations for ocean regimes shown in *SI Appendix*, Table S2 were applied if morphometric data and age were not reported.

### Data Analysis and Statistics.

In this study, data for BFT collected from 1998 to 2019 were evaluated, a time interval that is within the life span of BFT and which represents contemporary bioaccumulation status and exposure risk. MARs for BFT from each ocean basin were obtained as the slope of THg concentration versus age relationships for fish binned into 1-y age classes (*SI Appendix*, Table S3 by group and individual). Within each population, MARs for age-binned and unbinned values were nearly the same. MAR values were hence retrieved as the slopes for age-binned values as described below. Since the maximum age of BFT from the four populations studied varied from 9 y (MS) to 29 y (NAO), MARs were estimated for three groups of BFT, those ≤10 y old, those ≤15, and those of all ages (*SI Appendix*, Table S3)

In order to eliminate the variabilities of fish body and Hg levels in certain age ranges, we grouped values into age groups in increments of 1 y (11). The variability of the fish body may be due to their different growth rates at the same age or/and variable Hg levels due to variable preys they ate from vertical foraging depths within the same region (9). Least-square regressions of the BFT Hg concentration versus age were then performed (Fig. 2 and *SI Appendix*, Table S3). Regression slope values (i.e., MARs = ΔHg/Δy) of Hg versus age and average Hg concentrations of BFT across ocean regions were compared using a *t* test for their significant differences. This was finally followed by the one-way ANOVA to test differences of significance for regional effects. Relationships between MARs and the concentrations and column inventories of MeHg in seawater (surface ocean: 0 to 150 m; thermocline layer: 150 to 1,000 m) from the four ocean subbasins were shown in Figs. 3 and 4.

## Supplementary Material

Supplementary File

## Data Availability

All study data are included in the article and/or *SI Appendix*.
