# Supplementary material for: Bluefin tuna reveal global patterns of mercury pollution and bioavailability in the world's oceans
Source: Proc Natl Acad Sci U S A. 2021 Sep 13;118(38):e2111205118. doi: 10.1073/pnas.2111205118 (PMC8463802; doi:10.1073/pnas.2111205118)
Supplement: Supplementary File [file pnas.2111205118.sapp.pdf]

PNAS
www.pnas.org

Supplementary Information for

Bluefin Tuna Reveal Global Patterns of Mercury Pollution and Bioavailability in the World's Oceans

Chun-Mao Tseng[1*], Shin-Jing Ang[1], Yi-Sheng Chen[1], Jen- Chieh Shiao[1], Carl H. Lamborg[2], Xiao-Shuai He[3], John R. Reinfelder[3]

*Corresponding author: **Chun-Mao Tseng**

E-mail: cmtseng99@ntu.edu.tw

This PDF file includes

- Supplementary text
- Figures. S1 to S5
- Tables S1 to S8
- SI References

Supplementary text

Mercury analysis

Tuna THg concentrations were then measured using a flow injection THg analyzer (THg-FIMA), which is a dual purge-and-trap system combining aqueous reduction with stannous chloride ($SnCl_2$), two-stage gold (Au) amalgamation and thermal desorption together with cold vapor atomic fluorescence spectrometry. Briefly, aliquots of 50-100 µl of the digests were added to a gas-liquid separator (GLS) and diluted with Milli-Q water. An aliquot of 0.1 mL of the acidic $SnCl_2$ solution (20% w/v in HCl 12.5% v/v) was then added to reduce Hg^{2+} in solution to Hg^0. Generated Hg^0 was purged from the GLS vessel by Hg-free N_2 gas (6 minutes at 0.3 L min^{-1}) routing through a glass frit (~20 µm porosity) onto the Au-sand sample trap (ST), which was wrapped with a Ni-Cr wire attached to an adjustable voltage transformer for heating. The Hg^0 was then thermally desorbed (~600 °C) from the ST and onto an analytical trap (AT) and from that detected finally by the THg-FIMA detector (Tekran 2500) at an Ar flow of 30 mL min^{-1} for 4 min. The whole analytical process for Hg^0 analysis from sample delivery to final detection took 10 minutes and was automatically controlled by Quick Chrom (SISC Inc.) software. Overall, ~10 samples can be analyzed per hour with a two-channel detection of THg-FIMA. The THg-FIMA provides a system detection limit of 0.5 pg Hg, defined as three times the standard deviation of the procedural blank, with excellent reproducibility (RSD ≤ 5%, n=30) and calibration (r^2 >0.99, n=30).

The quality assurance of THg analysis in each analytical batch was validated through the proper quality control by data comparison of the two channels (r^2>0.98, n=150), and analyses of certified biological reference materials (TORT-2, lobster hepatopancreas, 0.270±0.001 µg Hg g^{-1} dry weight (d.w.)); IAEA-436, tuna fish, 4.19±0.36 µg g^{-1} d.w.). Recoveries of Hg from the two CRMs yielded on average 101±5% and 100±4% (n=20),

respectively. MeHg concentrations of selected samples were also measured using the analytical methodologies published in Tseng et al. *(1, 2)*. We found the proportion of MeHg relative to THg was 98±6% (n=20) in PBFT from the WPO, which confirmed that all fish Hg is essentially MeHg, similar to PBFT from the EPO (99±6%) *(3)* and ABFT form the MS (91±10%) *(4)*.

Evaluation of human health risk

The consumption guideline limits for eating fish to assess human exposure and risk of Hg are generally as follows: (1) fish tissue concentrations; (2) provisional tolerable daily or weekly intake (PTDI or PTWI). The concentration threshold of 1 μg Hg g^{-1} w.w. for BFT fish muscle tissue was, for instance, set by the World Health Organization (WHO) and European Commission (EC). THg levels considerably exceeding the recommended limit of 1 μg g^{-1} w.w. should be consumed with caution and even no consumption as suggested by the US EPA/FDA. As for the PTDI and PTWI, the reference doses (Rfd) for MMHg of 0.23 μg kg^{-1} day^{-1} and 1.6 μg kg^{-1} $week^{-1}$ were established by the US Environmental Protection Agency (US EPA) and WHO. Assuming that the concentration of THg is equal to that of MMHg and a body weight of 60 kg for an adult, the guideline values calculated from the Rfds of the US EPA are ~14 μg day^{-1} and ~98 μg $week^{-1}$, respectively. Based on the MAR of each ocean basin, we can calculate the size and age of fish to be avoided because threshold values will be exceeded, and how much fish we can safely eat and how often.

Table S1. **Bio-information of the Bluefin tuna** (e.g., species, distribution, size, age, Maturity/diet, diving depth, and spawning) on a global scale.

	Distribution	Max. length (cm)	Max. weight (kg)	Max. age (years)	Maturity/Diet	Diving depth* (m)	Spawning location/season
Pacific bluefin tuna (PBFT, *Thunnus orientalis)*	North Pacific Ocean (NPO)	300[a]	450[a]	30[a]	Reproduce later (5 years)/ Small fish (e.g., sardines, herring, mackerel), crustaceans, and invertebrates (e.g., squid).	Usually <200 m (600 m deeper)[b]	(1) West Pacific Ocean from April to June; (2) Kuroshio and the Kuroshio–Oyashio transition area from May to August; (3) Japan Sea from July to August[c]
Atlantic bluefin tuna (ABFT, Thunnus thynnus)	Atlantic Ocean (AO) and Mediterranean Sea (MS)	450[d]	680[d]	35[d]	Reproduce later (5 years)/ Small fish (e.g., sardines, herring, mackerel), crustaceans, and invertebrates (e.g., squid).	Usually <200 m (1000 m deeper)[e]	(1) Gulf of Mexico from April to June; (2) Mediterranean from June to July.[f]
Southern bluefin tuna (SBFT, Thunnus maccoyii)	South oceans (Indian Ocean/IO)	>220[d]	>200	40[g]	Reproduce later (5 years)/ Small fish (e.g., sardines, herring, mackerel), crustaceans, and invertebrates (e.g., squid).	Usually <200 m (1000 m deeper)[h]	Northeast Indian Ocean off northwest Australia from Sept. to April.[i]

a. Shimose et al. 2009 (5); b. Kitagawa et al. 2000 (6); c. Ohshimo et al. 2018 (7); d. Murua et al. 2017 (8); e. Block et al. 2001 (9); f. Aranda et al. 2013 (10); g. Gunn et l. 2008 (11); h. Patterson et al. 2008 (12); i. Farley et al. 2014 (13)

* BFT commonly spend >90% of their lifetime in waters shallower than 200 m.

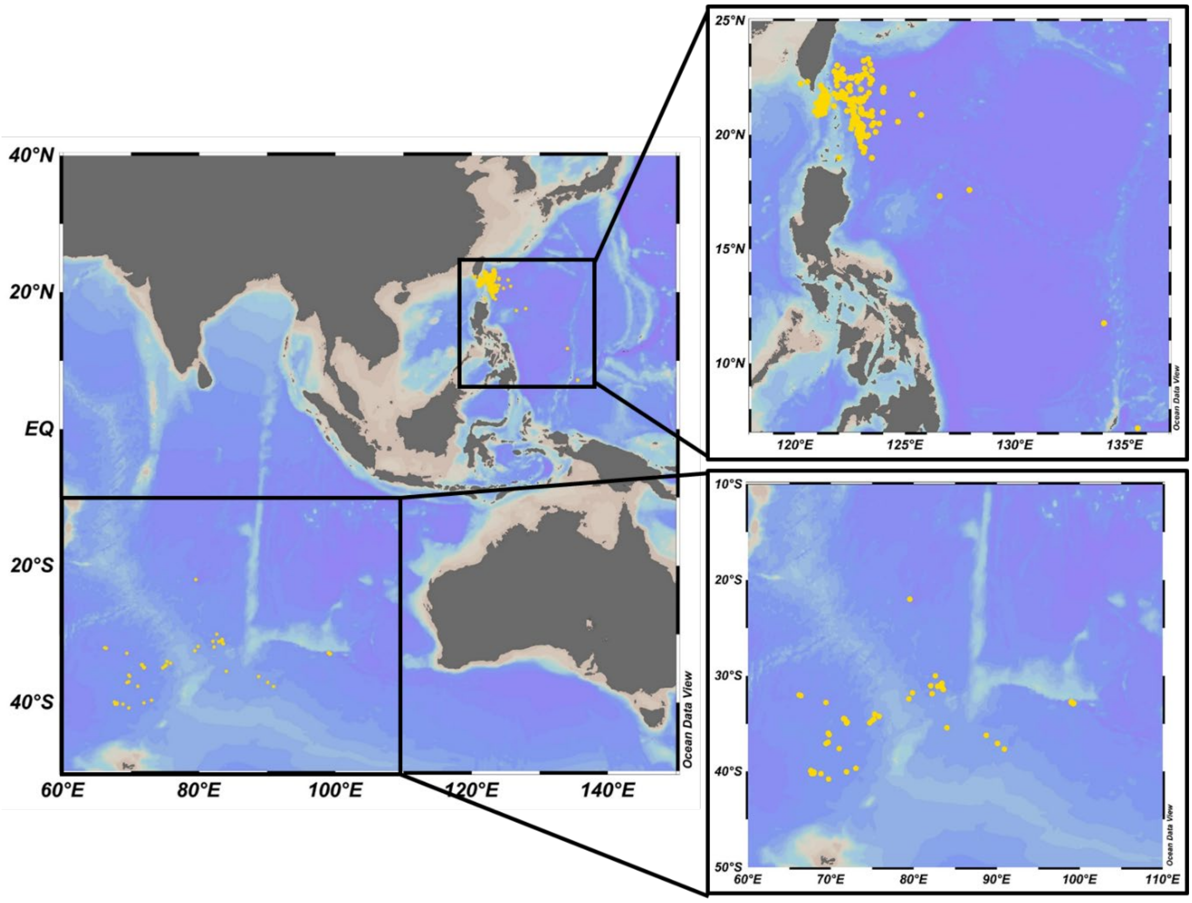

Fig. S1 **Sampling locations** for Pacific Bluefin tuna (Pacific BFT) captured off the east and southeast offshores of Taiwan and Southern Bluefin tuna (Southern BFT) from the southern hemisphere Indian Ocean.

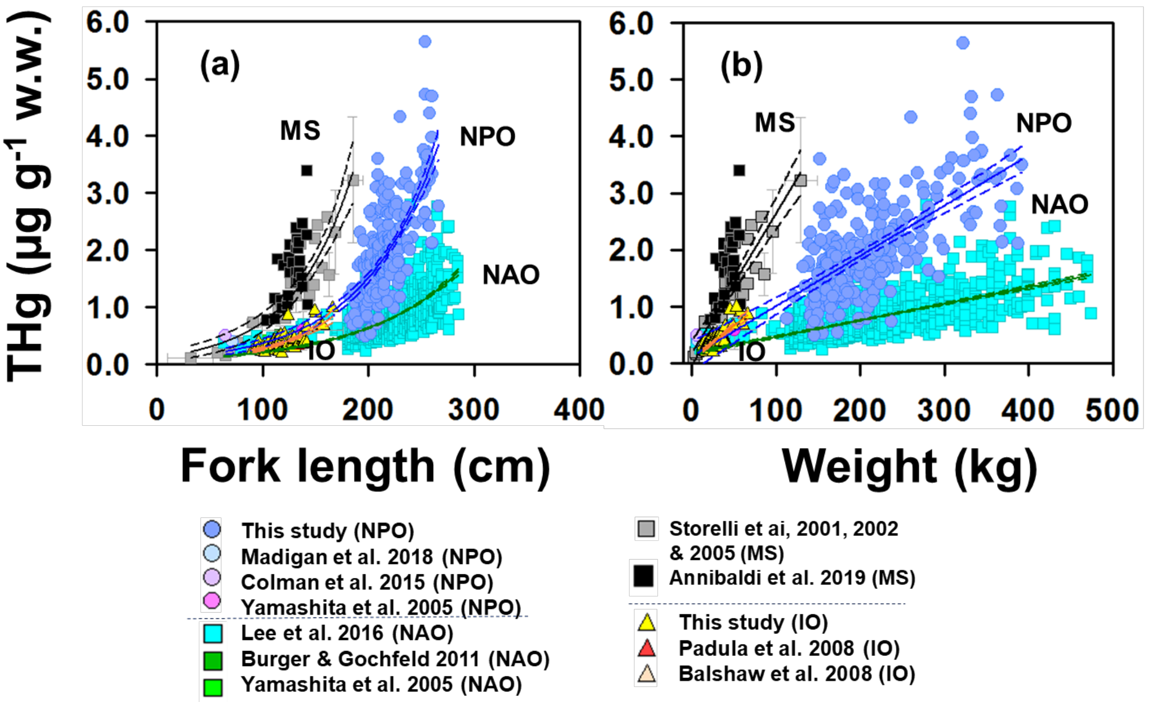

Fig. S2. **Positive relationships between BFT THg levels vs (a) fork length, and (b) fish weight among different ocean regions (See references in the main text).** Errors are ± 1 SD; regressions with the 95% confidence interval lines.

Table S2. Total Hg concentrations in the BFTs with sample information from global ocean basins collected from this study and the peer-review literature.

Species	Collection/Ocean	No.	FL (cm)	Weight (kg)	Age (yrs)	THg (µg/g w.w.)	References
Pacific BFT (*Thunnus orientalis*)	Taiwan/ NPO	261	216.9±18.6 (182-266)	211.8±61.2 (113-392)	11.5±6.3[a] (5.5-27.0)	2.00 ± 0.83 (0.49-5.65)	This study
	California/ NPO	24	71.2±3.3 (66−76)	7.6±1.0[b] (6.1-9.1)	1.5±0.1[c] (1.4-1.7)	0.39±0.11	Madigan et al. 2018
	California/ NPO	16	64.3±2.5[d] (61-68)	6.5±0.7[b] (5.5-7.4)	1.3±0.1[c] (1.2-1.4)	0.50±0.07	Colman et al. 2015
	Japan/ NPO	16	137.8±38.6[b] (48-162)	50.5±26.7 (2.5-81)	4.2±1.5[c] (0.8-5.3)	0.59±0.34	Yamashita et al. 2005
Atlantic BFT (*Thunnus thynnus*)	Gulfs of Maine & St. Lawrence /NAO	1292	210.6±28.3[e] (63-285)	199.1±79.5[f] (4.8-474.0)	12.3±3.8 (0.8−29.0)	0.76 ± 0.33 (0.25−3.15)	Lee et al. 2016
	New Jersey/ NAO	23	116.3±4.8[g] (66-144)	30.8±18.3[f] (5.5-59.3)	4.0±1.4[f] (1.5-5.7)	0.52±0.16	Burger and Gochfeld 2011
	Japan/ NAO	20	96.9±19.6[f] (68-126)	21.8±11.2 (6.0-39.0)	3.4±1.0[f] (1.6-4.6)	0.42±0.06	Yamashita et al. 2005
	Ionian Sea/ MS	73	56.7±2.7[h] (53-60)	3.6±0.5 (2.9–4.4)	1.1±0.1[h] (1.0-1.3)	0.20 ± 0.07 (0.13–0.35)	Storelli et al. 2005
	Ionian Sea/ MS	161	121.8±32.7[h] (64-161)	36.1±23.53 (5.3-83)	4.2±1.7[h] (1.5-6.6)	1.18±0.85 (0.16-2.59)	Storelli et al. 2002
	Thyrrenian Sea/MS	169	125.5±58.9[h] (26-199)	39.5±43.8 (0.3-158.0)	4.4±3.2[h] (0-9.5)	1.02±0.99 (0.07–4.26)	Storelli and Marcotrigiano 2001
	Sardinia/ MS	33	135±23 (104-142)	45±26 (22.3-57.3)	4.6±0.6[h] (3.3-5.4)	1.69±0.58 (0.78–3.40)	Annibaldi et al. 2019
Southern BFT (*Thunnus maccoyii*)	Indian Ocean/IO	83	119.5±16.2 (85-166)	28.0±12.8 (13-96)	5.3±2.3[j] (2.7-15.0)	0.42±0.24 (0.20-1.85)	This study
	Spencer Gulf/IO	5	105.0±4.1 (96-108)	23.0±3.5[i] (16.1-26.4)	3.7±0.3[j] (3.1-3.9)	0.34 (0.28–0.42)	Padula et al. 2008
	Great Australian Bight/IO	6	102.2±13.6 (88-122)	27.4±10.1 (16.2-41.6)	3.6±0.9[j] (2.6-5.0)	0.29±0.04	Balshaw et al. 2008
	Japan/IO	7	126.6±4.7[i] (121-134)	40.3±4.4 (35.0-48.0)	5.4±0.5[j] (4.9-6.2)	0.27±0.04	Yamashita et al. 2005

a. (14); b.(5); c. (15); d. (16); e. (17); f.(18); g. (19); h. (20); i. (21); j.(11)

Table S3 Regression information between the BFT ages and THg concentrations in the full size (age 1-29) and small size class fish (less than 10, and 15 year olds) with age-binned and individual data among ocean regions.

Ocean basins	Data sources	Age range	Data	a (Slope)	b (Intercept)	R^2	*p*
NAO	Lee et al. 2016 +Burger et al. 2011 +Yamashita et al. 2005	1-10	Group	0.031±0.006	0.340±0.032	0.74	<0.0001(12)
			Raw	0.030±0.005	0.296±0.044	0.08	<0.0001(482)
		1-15	Group	0.036±0.003	0.313±0.026	0.90	<0.0001(17)
			Raw	0.053±0.003	0.081±0.029	0.28	<0.0001(1083)
		1-29	Group	0.047±0.002	0.255±0.039	0.93	<0.0001(31)
			Raw	0.061±0.002	0.007±0.021	0.52	<0.0001(1276)
IO	This study + Padula et al. 2008 +Balshaw et al. 2008	1-10	Group	0.082±0.017	-0.003±0.102	0.73	0.002(9)
			Raw	0.065±0.007	0.107±0.036	0.42	<0.001(80)
		1-15	Group	0.110±0.014	-0.160±0.106	0.87	<0.001(11)
			Raw	0.096±0.008	-0.084±0.046	0.63	<0.001(85)
NPO	This study + Madigan et al. 2018 +Colman et al. 2015 +Yamashita et al. 2005	1-10	Group	0.225±0.017	0.056±0.113	0.94	<0.0001(9)
			Raw	0.239±0.027	-0.282±0.228	0.29	<0.0001(184)
		1-15	Group	0.170±0.011	0.167±0.100	0.95	<0.0001(14)
			Raw	0.165±0.017	0.292±0.157	0.29	<0.0001(225)
		1-29	Group	0.107±0.009	0.617±0.134	0.87	<0.0001(21)
			Raw	0.095±0.006	0.904±0.075	0.51	<0.0001(264)
MS	Storelli & Marcotrigiano 2001 + Storelli et al. 2002, 2005 +Annibaldi et al. 2019	1-9	Group	0.405±0.035	-0.904±0.095	0.93	<0.0001(11)
			Raw	0.429±0.041	-0.425±0.188	0.65	<0.0001(59)

(n): Number of individual or bins

Table S4 One-way analysis of variance (ANOVA) testing significant differences of variables among various ocean basins.

	DF	SS	MS	F	P
THg (μg g^{-1} w.w.)	3	273.523	91.174	248.94	<0.001***
MAR of age 1-10yrs (μg g^{-1} yr^{-1})	3	0.592	0.197	20.74	<0.001***
MAR of age 1-15yrs (μg g^{-1} yr^{-1})	3	0.702	0.234	35.23	<0.001***
MAR of age 1-30yrs (μg g^{-1} yr^{-1})	3	0.934	0.311	65.67	<0.001***
0-150 m THg (pM)	3	1.040	0.347	4.372	0.019**
150-1000 m THg (pM)	3	0.535	0.178	2.282	0.118*
0-1000 m THg inventory (μmol m^{-2})	3	0.675	0.225	3.632	0.025**
0-150 m MeHg (fM)	3	42120	14040	5.585	0.011**
150-1000 m MeHg (fM)	3	100649	33550	4.143	0.013**
0-1000 m MeHg inventory (nmol m^{-2})	3	132595	44198	7	<0.001***

DF: Degrees of freedom; SS: Sum-of-square; MS: Mean square; F: F ratio;
P: *P* values (Significant differences at 85%*, 95%**, and 99%*** confidence levels).

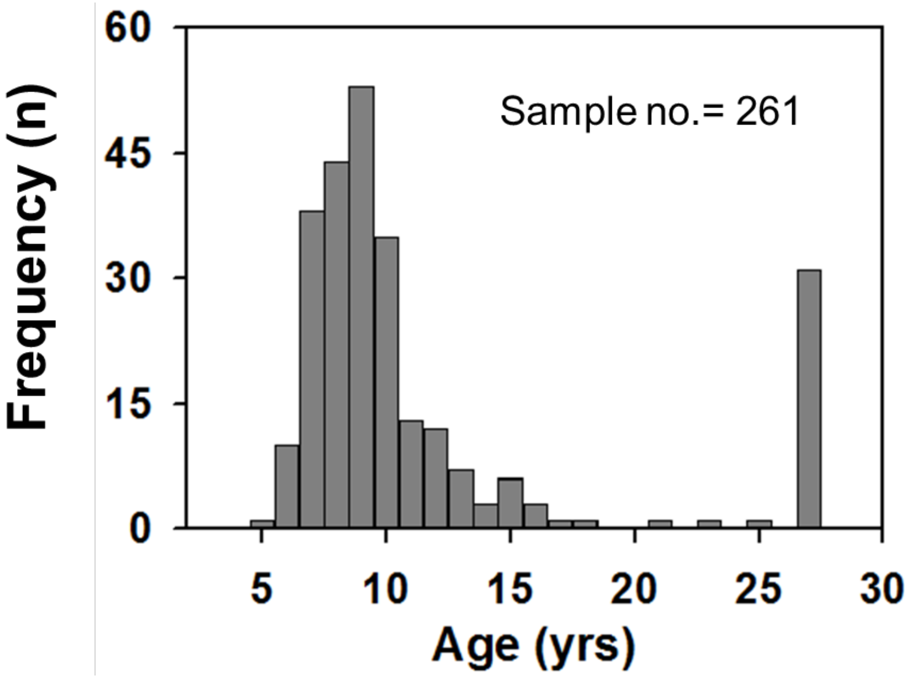

Fig. S3 Histograms of fish age in Pacific Bluefin tuna in 2017 and 2018.

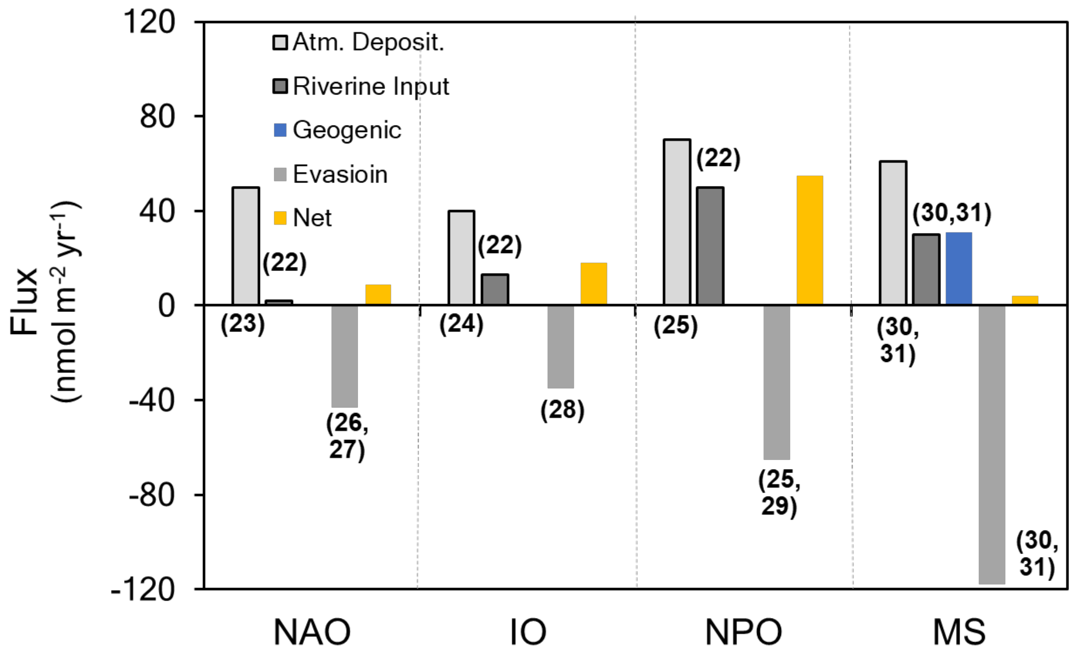

Fig. S4 **A simplified evaluation of the Hg budget in the upper waters of four ocean sub-basins, with citations shown in parentheses.** Riverine inputs include dissolved and particulate Hg portions reaching the open ocean (22).

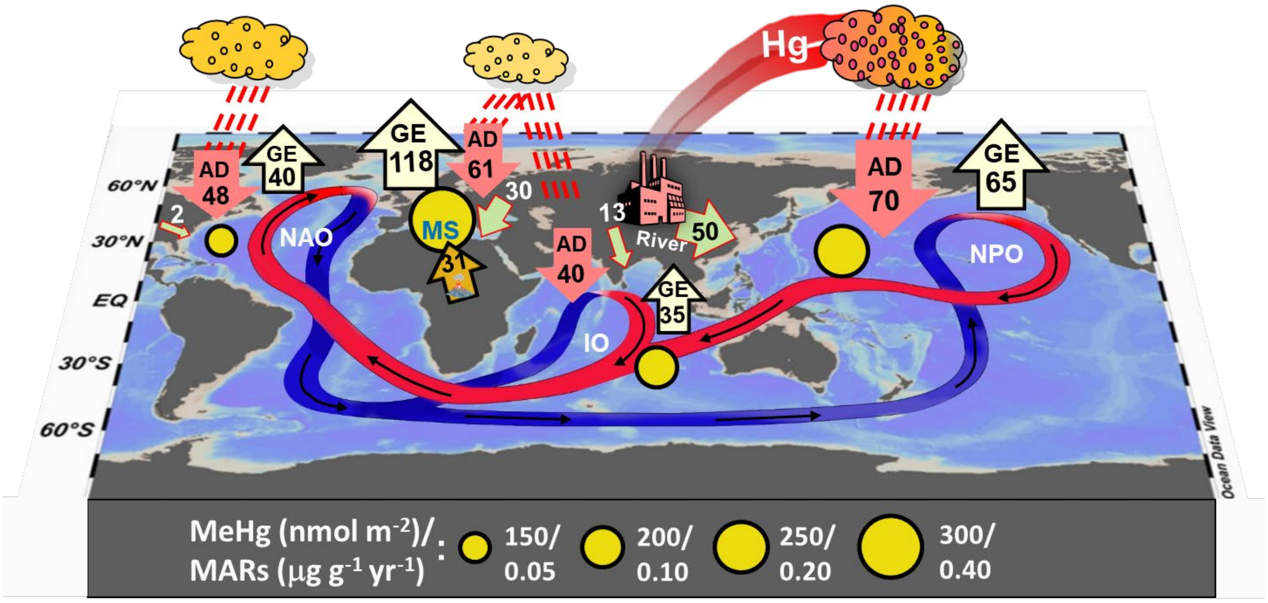

Fig. S5. **Conceptual model of mercury accumulation rates (MARs) in Bluefin tuna (circles) in relation to methylmercury (MeHg) inventories in the world's oceans and global patterns of Hg pollution with simplified Hg fluxes** (detailed information in Fig. S4). The NPO receives a net Hg input of over 55 nmol m^{-2} yr^{-1} from atmospheric deposition (AD, ~70 nmol m^{-2} yr^{-1}), river inputs (~50 nmol m^{-2} yr^{-1}), after the evasion of gaseous Hg (GE, ~65 nmol m^{-2} yr^{-1}). Net inputs of Hg to the IO and NAO are ~18 nmol m^{-2} yr^{-1} and ~10 nmol m^{-2} yr^{-1}, respectively. Upwelled MeHg from the western equatorial Pacific Ocean may enter the IO via the Indonesian Throughflow, but little such MeHg is expected to return to the NAO. The MS receives large inputs of Hg from atmospheric deposition, riverine inputs geogenic activities (~120 nmol m^{-2} yr^{-1}), which are approximately equal to gaseous evasion and its oligotrophic ecology may support higher levels of MeHg production compared to other ocean basins although a net flux ~zero needed to be examined in near future.

Table S5 **Concentrations and bioaccumulation factors of MeHg in marine plankton among ocean regions.**

Basins	Coordinates	Phytoplankton MeHg (pmol g^{-1} w.w.)	Phytoplankton %MeHg	Phytoplankton Log BAF* (L Kg^{-1})	Zooplankton MeHg (pmol g^{-1} w.w.)	Zooplankton %MeHg	Zooplankton Log BAF (L Kg^{-1})	References
NAO	NW Atlantic	0.7±0.31 (0.11–1.3)	6±3	4.3±0.3	2.8±2.8	15±12	4.9	Hammerschmidt et al. 2013 *(32)*
NAO	NE Atlantic -Bay of Biscay	-	-	-	1.9±0.7	33±12	4.8	Cossa et al. 2012 *(33)*
NPO	NE Pacific-SAFe site (30°N–50°N, 140°W)	4	-	5.2	-	-	-	Hammerschmidt and Bowman, 2012 *(34)*
NPO	Central Pacific	2.9±2.6 (0.5–8.2)	10 (3-29)	5.2 (4.4-6.2)	4 (1-17)	16 (13-20)	5.3 (4.1-6.5)	Gosnell and Mason, 2015 *(35)*
MS	Mediterranean Sea (Gulf of Lions) (41°N–42°N, 4.5°E-6°E)	-	-	-	6.1±6.9	12±13	5.4	Cossa et al. 2012 *(33);*

*. BAF denotes bioaccumulation factor i.e., MeHg concentration (w.w.) in organisms divided by that in associated filtered water.

Table S6 Hg accumulation rates (MARs) in different sub-ocean basins with the information of environmental factors

Ocean basins	MARs (μg g^{-1} yr^{-1})	MeHg (fM) (nmol m^{-2})			Phytoplankton		Zooplankton		Growth rate	Migration Main pattern	Water temp. range	Diet/Prey[g]
		0-150 m	150-1000 m	0-1000 m	MeHg (pmol g^{-1})	Log BAF (L Kg^{-1})	MeHg (pmol g^{-1})	Log BAF (L Kg^{-1})	K (1 yr^{-1})[a]	(Latitude)	(^{0}C)	
NAO	0.04±0.011	49±16	153±99	130	0.7±0.3	4.3±0.3	2.4±2.4	4.9	0.089[b]	20-50 ^{0}N	3-30 (14-20)[f]	Small fish crustaceans, and invertebrates (e.g., squid).
IO	0.11±0.01	75±26	175±48	163	-	-	-	-	0.185[c]	20-50 ^{0}S	3-30 (18-20)	Same as above
NPO	0.17±0.01	89±75	261±95	211±69	3.5±2.6	5.2	4.0	5.3	0.17[d]	20-50 ^{0}N	3-30 (14-20)	Same as above
MS	0.41±0.04	190±68	312±55	294±49	-	-	6.1±6.9	5.4	0.093[e]	30-40 ^{0}N	3-30	Same as above

[a] k: von Bertalanffy growth coefficient; [b] (18); [c] (11); [d] (14, 15); [e] (20)

[f] Preferred temperature range

[g] Diet composition is variable since bluefin tuna engage diverse food habit in opportunistic feeding

Table S7 A brief evaluation of the Hg budget in the Mediterranean

	Rajar et al., 2007 (30)		Zagar et al., 2014 (31)		Average
	[a]Mmol yr^{-1}	[b]nmol m^{-2} yr^{-1}	[a]Mg yr^{-1}	nmol m^{-2} yr^{-1}	nmol m^{-2} yr^{-1}
Atmospheric deposition	0.115	46	38	75.8	60.9
River inputs[c] (+Point sources)	0.065 (+0.0125)	26 (+5)	13 (+2.5)	25.9 (+5)	26 (+5)
Oceanic evasion	0.25	100	68	135.6	117.8
Geogenic sources	0.08	32.0	15	29.9	31.0

[a] Mmol yr^{-1} (Megamoles per year); Mg yr^{-1} (megagrams per year)

[b] All numbers are derived from the surface area of the Mediterranean, ca. 2.5×10^6 km^2

[c] Riverine inputs if considered the fraction of particulate Hg reaching to open marine waters (~16% for the MS) maybe near 5 nmol m^{-2} yr^{-1}, similar to that proposed by Amos et al., 2014 (22).

Table S8 **Total Hg and MeHg[a] concentrations in the seawaters of surface and thermocline layers with their inventories in ocean regimes.**

Basins	Coordinates	THg (pM)		MeHg (fM)		THg (μmol m^{-2})	MeHg (nmol m^{-2})	References
		0-150 m	150-1000 m	0-150 m	150-1000 m	0-1000 m	0-1000 m	
IO	Southern Atlantic Ocean (40° S, 50° W-10° E)	1.45±0.60		56.5	-	-	-	Bratkič et al. 2016 *(36)*
	Southern Ocean (44.0° S-52.7° S, 140° E)	1.19±0.27	1.15±0.22	93±35	175±48	1.16	163	Cossa et al. 2011 *(37)*[b]
	**Mean**	**1.19±0.27**	**1.15±0.22**	**75±26**	**175±48**	**1.16**	**163**	
NAO	North Atlantic Ocean	-	0.94±0.27	-	-	0.94	-	Lamborg et al. 2014 *(38)*
	North Atlantic-GA03 (22°N–40°N, 10° W-70° W)	0.65±0.32	-	60±50	-	0.65	-	Bowman et al. 2015 *(39)*
	North Atlantic-A16N (58°N–61.6°N, 20° W)	0.50±0.06	0.60±0.06	38±18	153±99	0.58	130	Soerensen et al. 2016 *(40)*
	**Mean**	**0.58±0.11**	**0.77±0.24**	**49±16**	**153±99**	**0.74±0.23**	**130±40**	
NPO	Northeast Pacific Ocean-P16N (22.5°N–50°N, 152°W)	0.99±0.32	1.35±0.37	95±52	260±114	1.30	235	Sunderland et al. 2009 *(41)*
	Northeast Pacifc-SAFe (30°N–50°N, 140°W)	0.37±0.10	0.78±0.17	24±3	98±18	0.72	87	Hammerschmidt and Bowman 2012 *(42)*[b]
	California coast (34°N–45°N, 157.5-119°W)	1.12±0.91	1.08±0.40	113±26	279±68	1.09	254	Coale et al. 2018 *(43)*[b]
	North Pacific Ocean-ALOHA (22.45°N, 158°W)	0.62±0.20	1.70±0.82	73±41	194±77	1.54	176	Motta et al. 2019 *(44)*[b]
	Western North Pacific (31° S−51°N, 129−171° E)	0.67 ± 0.18	1.1 ± 0.60	23 ± 30	328 ± 271	1.13	267	Kim et al. 2017 *(45)*

	North Pacific Ocean (22°N-50°N, 147 °E-158 °W)	1.15±0.86	1.4 ± 0.37	170	-	1.28	247	Laurier et al. 2004 *(46)*
	**Mean**	**0.82±0.31**	**1.24±0.32**	**83±56**	**232±89**	**1.17±0.27**	**211±69**	
MS	Mediterranean Sea (32.7°N–42°N, 06°E-28°E)	1.47 ± 0.41	1.83 ± 0.19	280 ± 49	300 ± 13	1.78	297	Horvat et al. 2003 *(47)*[b]
	Mediterranean Sea (35°N–43°N, 5°E-19°E)	0.99 ± 0.35	1.12 ± 0.34	116 ± 53	260 ± 82	1.10	238	Cossa et al. 2009 *(48)*[b]
	Mediterranean-Gulf of Lions (41°N–42°N, 4.5°E-6°E)	-	-	192 ± 89	299 ± 53	-	283	Cossa et al. 2017 *(49)*[b]
	Mediterranean-Ligurian basin (43.4°N, 8°E)	-	-	170 ± 110	390 ± 140	-	357	Heimburger et al. 2010 *(50)*[b]
	**Mean**	**1.23±0.34**	**1.48±0.50**	**190±68**	**312±55**	**1.44±0.48**	**294±49**	

[a] MeHg here denotes total methylated concentrations, including monomethyl- and dimethyl-Hg since some analytical methods used can't separate both species.

[b] Depth-weighted means calculated from the vertical profiles published in the literature

References

1. C. M. Tseng, A. De Diego, F. M. Martin, D. Amouroux, O. F. Donard, Rapid determination of inorganic mercury and methylmercury in biological reference materials by hydride generation, cryofocusing, atomic absorption spectrometry after open focused microwave-assisted alkaline digestion. *J. Anal. At. Spectrom.* **12**, 743-750 (1997).
2. C.-M. Tseng, C. R. Hammerschmidt, W. F. Fitzgerald, Determination of methylmercury in environmental matrixes by on-line flow injection and atomic fluorescence spectrometry. *Anal. Chem.* **76**, 7131-7136 (2004).
3. D. J. Madigan *et al.*, Mercury stable isotopes reveal influence of foraging depth on mercury concentrations and growth in Pacific bluefin tuna. *Environ. Sci. Technol.* **52**, 6256-6264 (2018).
4. M. Storelli, R. G. Stuffler, G. Marcotrigiano, Total and methylmercury residues in tuna-fish from the Mediterranean Sea. *Food Addit. Contam.* **19**, 715-720 (2002).
5. T. Shimose, T. Tanabe, K.-S. Chen, C.-C. Hsu, Age determination and growth of Pacific bluefin tuna, Thunnus orientalis, off Japan and Taiwan. *Fish. Res.* **100**, 134-139 (2009).
6. T. Kitagawa *et al.*, Effect of ambient temperature on the vertical distribution and movement of Pacific bluefin tuna *Thunnus thynnus orientalis*. *Mar. Ecol. Prog. Ser.* **206**, 251-260 (2000).
7. S. Ohshimo *et al.*, Evidence of spawning among Pacific bluefin tuna, Thunnus orientalis, in the Kuroshio and Kuroshio–Oyashio transition area. *Aquat. Living Resour.* **31**, 33 (2018).
8. H. Murua, E. Rodriguez-Marin, J. D. Neilson, J. H. Farley, M. J. Juan-Jordá, Fast versus slow growing tuna species: age, growth, and implications for

population dynamics and fisheries management. *Rev. Fish Biol. Fish.* **27**, 733-773 (2017).

9. B. A. Block *et al.*, Migratory movements, depth preferences, and thermal biology of Atlantic bluefin tuna. *Science* **293**, 1310-1314 (2001).
10. G. Aranda, F. J. Abascal, J. L. Varela, A. Medina, Spawning behaviour and post-spawning migration patterns of Atlantic bluefin tuna (*Thunnus thynnus*) ascertained from satellite archival tags. *PLoS One* **8**, e76445 (2013).
11. J. S. Gunn, *et al.,* Age and growth in southern bluefin tuna, *Thunnus maccoyii* (Castelnau): direct estimation from otoliths, scales and vertebrae. *Fish. Res.* **92**, 207-220 (2008).
12. T. A. Patterson, K. Evans, T. I. Carter, J. S. Gunn, Movement and behaviour of large southern bluefin tuna (*Thunnus maccoyii*) in the Australian region determined using pop-up satellite archival tags. *Fish. Oceanogr.* **17**, 352-367 (2008).
13. J. H. Farley *et al.*, Demographic structure, sex ratio and growth rates of southern bluefin tuna (*Thunnus maccoyii*) on the spawning ground. *PLoS One* **9**, e96392 (2014).
14. J.-C. Shiao *et al.*, Changes in size, age, and sex ratio composition of Pacific bluefin tuna (*Thunnus orientalis*) on the northwestern Pacific Ocean spawning grounds. *ICES J. Mar. Sci.* **74**, 204-214 (2016).
15. W. H. Bayliff, Growth and age composition of northern bluefin tuna, Thunnus thynnus, caught in the eastern Pacific Ocean, as estimated from length-frequency data, with comments on trans-Pacific migrations. *IATTC. Bull.* **20**, 501-540 (1993).
16. E. E. Estess *et al.*, Bioenergetics of captive Pacific bluefin tuna (Thunnus

orientalis). *Aquaculture* **434**, 137-144 (2014).

17. D. H. Secor *et al.*, Conversion factors for Atlantic bluefin tuna fork length from measures of snout length and otolith mass. *ICCAT Collected Volume of Scientific Papers* **70**, 364-367 (2014).
18. J. L. Cort, V. D. Estruch, Analysis of the Length–Weight Relationships for the Western Atlantic Bluefin Tuna, *Thunnus thynnus* (L.). *Rev. Fish. Sci. Aquac.* **24**, 126-135 (2016).
19. R. Froese, D. Pauly (FishBase [online]. Mumbai: FishBase Team; 2019 [cited 2019 Feb 10].
20. J. L. Cort *et al.*, On the Variability of the Length–Weight Relationship for Atlantic Bluefin Tuna, *Thunnus thynnus* (L.). *Rev. Fish. Sci. Aquac.* **23**, 23-38 (2015).
21. E. Bubner, J. Farley, P. Thomas, T. Bolton, A. Elizur, Assessment of reproductive maturation of southern bluefin tuna (*Thunnus maccoyii*) in captivity. *Aquaculture* **364**, 82-95 (2012).
22. H. M. Amos et al., Global biogeochemical implications of mercury discharges from rivers and sediment burial. *Environ. Sci. Technol.* **48**, 9514-9522 (2014).
23. S. W. Gichuki, R. P. Mason, Wet and dry deposition of mercury in Bermuda. *Atmos. Environ.* **87**, 249-257 (2014).
24. S. W. Gichuki, R. P. Mason, Mercury and metals in South African precipitation. *Atmos. Environ.* **79**, 286-298 (2013).
25. F. J. Laurier, R. P. Mason, L. Whalin, S. Kato, Reactive gaseous mercury formation in the North Pacific Ocean's marine boundary layer: A potential role of halogen chemistry. *J. Geophys. Res. Atmos.* **108,** D17 (2003).
26. R. P. Mason, C. R. Hammerschmidt, C. H. Lamborg, K. L. Bowman, G. J. Swarr,

R. U. Shelley, The air-sea exchange of mercury in the low latitude Pacific and Atlantic Oceans. *Deep Sea Res. I* **122**, 17-28 (2017).

27. J. Kuss, C. Zülicke, C. Pohl, B. Schneider, Atlantic mercury emission determined from continuous analysis of the elemental mercury sea-air concentration difference within transects between 50° N and 50° S. *Global Biogeochem. Cycles* **25**, GB3021 (2011).
28. A. L. Soerensen, E. M. Sunderland, C. D. Holmes, D. J. Jacob, R. M. Yantosca, H. Skov, J.H. Christensen, S.A. Strode, R. P. Mason, An improved global model for air-sea exchange of mercury: High concentrations over the North Atlantic. *Environ. Sci.Technol.* **44**, 8574-8580 (2010).
29. A. L. Soerensen, R. P. Mason, P. H. Balcom, D. J. Jacob, Y. Zhang, J. Kuss, E. M. Sunderland, Elemental mercury concentrations and fluxes in the tropical atmosphere and ocean. *Environ. Sci. Technol.* **48**, 11312-11319 (2014).
30. R. Rajar, M. Četina, M. Horvat, D. Žagar, Mass balance of mercury in the Mediterranean Sea. *Mar. Chem.* **107**, 89-102 (2007).
31. D. Žagar *et al.,* Mercury in the Mediterranean. Part 2: processes and mass balance. **21**, 4081-4094 (2014).
32. C. R. Hammerschmidt, M. B. Finiguerra, R. L. Weller, W. F. Fitzgerald, Methylmercury accumulation in plankton on the continental margin of the Northwest Atlantic Ocean. *Environ. Sci. Technol.* **47**, 3671-3677 (2013).
33. D. Cossa *et al.*, Influences of bioavailability, trophic position, and growth on methylmercury in hakes (*Merluccius merluccius*) from Northwestern Mediterranean and Northeastern Atlantic. *Environ. Sci. Technol.* **46**, 4885-4893 (2012).
34. C. R. Hammerschmidt, K. L. Bowman, Vertical methylmercury distribution in

the subtropical North Pacific Ocean. *Mar. Chem.* **132**, 77-82 (2012).

35. K. J. Gosnell, R. P. Mason, Mercury and methylmercury incidence and bioaccumulation in plankton from the central Pacific Ocean. *Mar. Chem.* **177**, 772-780 (2015).
36. A. Bratkič *et al.*, Mercury presence and speciation in the South Atlantic Ocean along the 40° S transect. *Global Biogeochem. Cycles* **30**, 105-119 (2016).
37. D. Cossa *et al.*, Mercury in the southern ocean. *Geochim. Cosmochim. Acta* **75**, 4037-4052 (2011).
38. C. H. Lamborg *et al.*, A global ocean inventory of anthropogenic mercury based on water column measurements. *Nature* **512**, 65 (2014).
39. K. L. Bowman, C. R. Hammerschmidt, C. H. Lamborg, G. Swarr, Mercury in the North Atlantic Ocean: The US GEOTRACES zonal and meridional sections. *Deep Sea Res. (II Top. Stud. Oceanogr.)* **116**, 251-261 (2015).
40. A. L. Soerensen *et al.*, A mass budget for mercury and methylmercury in the Arctic Ocean. *Global Biogeochem. Cycles* **30**, 560-575 (2016).
41. E. M. Sunderland, D. P. Krabbenhoft, J. W. Moreau, S. A. Strode, W. M. Landing, Mercury sources, distribution, and bioavailability in the North Pacific Ocean: Insights from data and models. *Global Biogeochem. Cycles* **23**, GB2010 (2009).
42. C. R. Hammerschmidt, K. L. Bowman, Vertical methylmercury distribution in the subtropical North Pacific Ocean. *Mar. Chem.* **132**, 77-82 (2012).
43. K. Coale *et al.*, The distribution and speciation of mercury in the California current: Implications for mercury transport via fog to land. *Deep Sea Res. (II Top. Stud. Oceanogr.)* **151**, 77-88 (2018).
44. L. C. Motta *et al.*, Mercury cycling in the North Pacific Subtropical Gyre as revealed by mercury stable isotope ratios. *Global Biogeochem. Cycles* (2019).

45. H. Kim *et al.*, Methylmercury mass budgets and distribution characteristics in the Western Pacific Ocean. *Environ. Sci. Technol.* **51**, 1186-1194 (2017).

46. F. Laurier, R. Mason, G. a. Gill, L. Whalin, Mercury distributions in the North Pacific Ocean—20 years of observations. *Mar. Chem.* **90**, 3-19 (2004).

47. M. Horvat *et al.*, Speciation of mercury in surface and deep-sea waters in the Mediterranean Sea. *Atmos. Environ.* **37**, 93-108 (2003).

48. D. Cossa, B. Averty, N. Pirrone, The origin of methylmercury in open Mediterranean waters. *Limnol. Oceanogr.* **54**, 837-844 (2009).

49. D. Cossa *et al.*, The open sea as the main source of methylmercury in the water column of the Gulf of Lions (Northwestern Mediterranean margin). *Geochim. Cosmochim. Acta* **199**, 222-237 (2017).

50. L.-E. Heimbürger *et al.*, Methyl mercury distributions in relation to the presence of nano-and picophytoplankton in an oceanic water column (Ligurian Sea, North-western Mediterranean). *Geochim. Cosmochim. Acta* **74**, 5549-5559 (2010).
